# Fluctuating Helical Asymmetry and Morphology of Snails (Gastropoda) in Divergent Microhabitats at ‘Evolution Canyons I and II,’ Israel

**DOI:** 10.1371/journal.pone.0041840

**Published:** 2012-07-26

**Authors:** Shmuel Raz, Nathan P. Schwartz, Hendrik K. Mienis, Eviatar Nevo, John H. Graham

**Affiliations:** 1 Department of Evolutionary and Environmental Biology, Institute of Evolution, University of Haifa, Haifa, Israel; 2 Rowland Institute at Harvard, Harvard University, Cambridge, Massachusetts, United States of America; 3 Department of Biology, Berry College, Mount Berry, Georgia, United States of America; 4 The Steinhardt National Collections of Natural History, Tel Aviv University, Tel Aviv, Israel; National Cancer Institute, United States of America

## Abstract

**Background:**

Developmental instability of shelled gastropods is measured as deviations from a perfect equiangular (logarithmic) spiral. We studied six species of gastropods at ‘Evolution Canyons I and II’ in Carmel and the Galilee Mountains, Israel, respectively. The xeric, south-facing, ‘African’ slopes and the mesic, north-facing, ‘European’ slopes have dramatically different microclimates and plant communities. Moreover, ‘Evolution Canyon II’ receives more rainfall than ‘Evolution Canyon I.’

**Methodology/Principal Findings:**

We examined fluctuating asymmetry, rate of whorl expansion, shell height, and number of rotations of the body suture in six species of terrestrial snails from the two ‘Evolution Canyons.’ The xeric ‘African’ slope should be more stressful to land snails than the ‘European’ slope, and ‘Evolution Canyon I’ should be more stressful than ‘Evolution Canyon II.’ Only *Eopolita protensa jebusitica* showed marginally significant differences in fluctuating helical asymmetry between the two slopes. Contrary to expectations, asymmetry was marginally greater on the ‘European’ slope. Shells of *Levantina spiriplana caesareana* at ‘Evolution Canyon I,’ were smaller and more asymmetric than those at ‘Evolution Canyon II.’ Moreover, shell height and number of rotations of the suture were greater on the north-facing slopes of both canyons.

**Conclusions/Significance:**

Our data is consistent with a trade-off between drought resistance and thermoregulation in snails; *Levantina* was significantly smaller on the ‘African’ slope, for increasing surface area and thermoregulation, while *Eopolita* was larger on the ‘African’ slope, for reducing water evaporation. In addition, ‘Evolution Canyon I’ was more stressful than Evolution Canyon II’ for *Levantina*.

## Introduction

Fluctuating asymmetry, a measure of developmental instability [1], is usually estimated from bilaterally symmetrical traits. Many organisms, however, have other kinds of symmetry (i.e., translatory, radial, dihedral, or helical symmetries). Gastropods, for example, have helical symmetry, which can be the basis for fluctuating helical asymmetry. Previously, Graham, Freeman & Emlen [2] studied deviations from a perfect equiangular (logarithmic) spiral in three populations of the terrestrial snail *Cepaea nemoralis* (Helicidae: Gastropoda) in the Ukraine. Others have studied shell deformities in snails [3] and other mollusks [4], [5], [6]. Here we study growth, shell morphology, and fluctuating helical asymmetry of six species of terrestrial pulmonate and prosobranch snails from the opposing slopes of ‘Evolution Canyon I,’ Lower Nahal Oren, Mount Carmel (EC I) and ‘Evolution Canyon II,’ Lower Nahal Keziv, Western Upper Galilee (EC II), in Israel.

The ‘Evolution Canyon’ microsites are model systems for the study of adaptation and speciation. The opposite slopes of these canyons, the abiotically stressed south-facing, ‘African’ slopes and the moderate, north-facing, ‘European’ slopes, diverge biotically and abiotically, providing an opportunity to study developmental instability in a natural experiment. Hundreds of studies have been conducted here in the last 20 years [7], [8], [9], [10], [11], [12].

**Figure 1:**
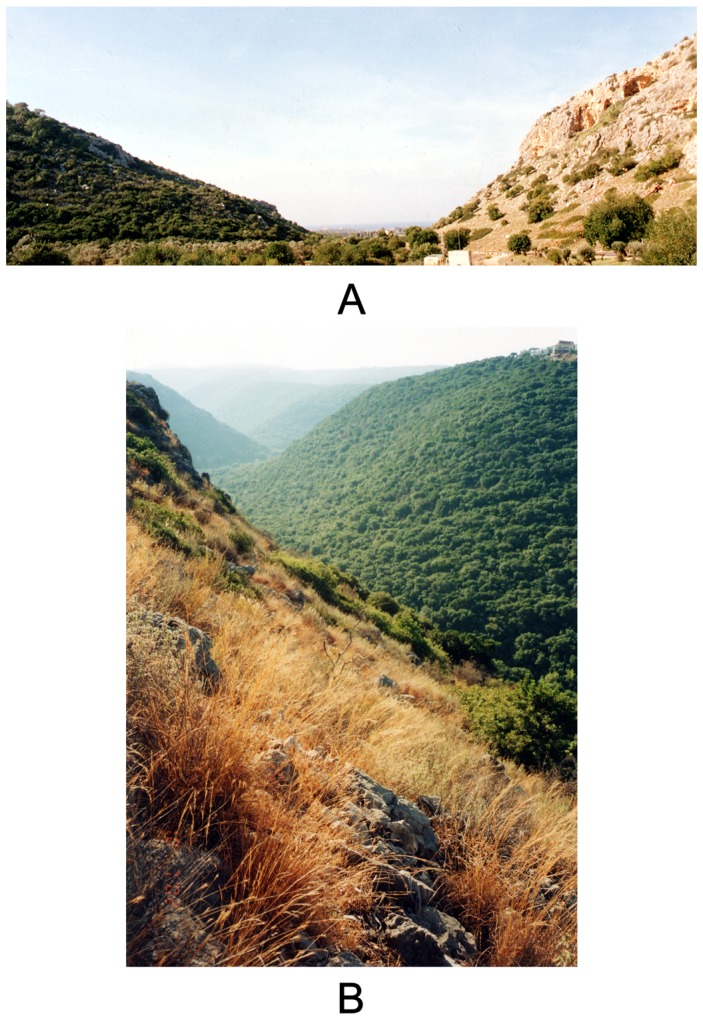
The opposing slopes of the ‘Evolution Canyons.’ The xeric ‘African’ slopes are on the right, and the mesic ‘European’ slopes are on the left. A. ‘Evolution Canyon I,’ Lower Nahal Oren, Mount Carmel, Israel. B. ‘Evolution Canyon II,’ Lower Nahal Keziv, western Upper Galilee, Israel.

Four ‘Evolution Canyon’ microsites are distributed across Israel: EC I in the mountains of Carmel, EC II in Galilee, EC III in the Negev, and EC IV in the Golan [10], [11]. Most of the studies on these canyons were conducted at ‘Evolution Canyons I and II’ (Figure 1). They have demonstrated that the ‘African’ slope is more stressful for many mesic organisms (reviewed in [7], [8], [9], [10], [11], [12]). The microclimatic differences produce strong differentiation of local biodiversity at all developmental levels (base sequences, genes, genomes, populations, species, ecosystems, and biota). The interslope differences at the molecular level (greater mutation frequency and recombination rate on the ‘African’ slope, in different taxa) are accompanied by interslope differences in species richness and abundance (reviewed in [7], [8], [9], [10], [11], [12]).

In 11 of 14 model species at ‘Evolution Canyon I,’ Nevo and colleagues [7], [8], [9], [10], [11], [12] found significantly greater genetic polymorphism on the ‘African’ slope than on the ‘European’ slope. They also found adaptive changes in other genetic characteristics. Populations of several model species on the more stressful ‘African’ slope had greater rates of mutation, gene conversion, recombination, and DNA repair, as well as greater genome size, more SSRs, SNPs, retrotransposons, transposons, candidate gene diversity, and genome-wide gene expression and regulation.

The coprophilous fungus *Sordaria fimicola*, for example, has heritable mutation rates 3-fold higher on the ‘African’ slope. *Drosophila melanogaster* has male recombination rates 4-fold higher on the ‘African’ slope. The filamentous cyanobacterium, *Nostoc linckia*, has higher haplotype diversity of clock genes *KaiABC* on the ‘African’ slope, and wild barley, *Hordeum spontaneum*, shows genetic divergence between the opposing slopes.

In addition to genetic divergence, species richness and abundance differ between the slopes. Pavlíček *et al.* [13], for example, showed that different taxonomic groups of terrestrial animals, such as scorpions, reptiles, butterflies (Rhopalocera), darkling beetles (Tenebrionidae), skin beetles (Dermestidae), and grasshoppers (Orthoptera), are more abundant on the ‘African’ slope than on the ‘European’ slope. The opposite trend occurs with springtails (Collembola), soil microfungi, basidiomycetous fungi (Basidiomycetes), mosses (Bryophyta), and trees and shrubs. These taxa have greater species richness on the ‘European’ slope than on the ‘African’ slope.

These results [13] demonstrate that species richness and abundance vary along a climatic transect of only a few hundred meters (the geology is identical on both slopes), revealing ecological (climatic) selection strong enough to override the mixing effects of migration and stochasticity. The ecological selection caused by higher insolation on the ‘African’ slope leads to greater ecological heterogeneity on that slope, as well as a savanna ecosystem that accommodates more species of heat-dependent taxa.

In a previous study at ‘Evolution Canyon I,’ Raz *et al.* [14] studied leaf asymmetry of twelve species of vascular plants growing on the opposing slopes. Two of the species had more asymmetrical leaves on the ‘African’ slope, while one species had more asymmetrical leaves on the ‘European’ slope. Overall, the differences in fluctuating asymmetry between the slopes were negatively correlated with the differences in relative abundance. Species displayed greater fluctuating asymmetry on the slope where they were less abundant, and hence more stressed.

In the current study we explore the fluctuating helical asymmetry of six land-snail species from the opposing slopes of ‘Evolution Canyons I and II.’ Because land snails are susceptible to desiccation, the xeric ‘African’ slope should be more stressful. But unlike plants, land-snails can hide under stones and in cracks. And like plants, they can also become inactive during the dry season. In Israel's Mediterranean region, snails are typically active during rainy days from November to April [15]. Such behavior reduces temperature stress and water loss [16]. Morphology, physiology, and life history can also influence resistance to desiccation [17]. Consequently, some species of land snails can live abundantly in deserts.

Previous research on snails at ‘Evolution Canyon I’ shows that most snails are larger on the ‘European’ slope, but more abundant on the ‘African’ slope [13], [15]. Rainfall is roughly the same on both slopes, but temperatures on the ‘African’ slope may be more amenable for growth during the rainy winter, when the snails are active. Broza & Nevo [15] suggested that the size differences between the two slopes might be due to *r*- and *k*-selection; snails on the ‘African’ slope put more energy into reproduction, while those on the ‘European’ slope put more energy into competitive ability. Size differences could also reflect Bergmann's ecogeographic rule extended to invertebrates: smaller body size supporting thermal tolerance on the warmer slope.

These results suggest that the interslope differences in insolation, temperature, and humidity at ‘Evolution Canyon’ differentially influence growth, morphology, and developmental instability of snails [18]. Hence, snails should be larger and more symmetrical on the ‘European’ slope than on the ‘African’ slope of ‘Evolution Canyon.’ We recognize, however, that the cool and humid ‘European’ slope could be stressful to land-snails adapted to more xeric and warm climatic conditions. Moreover, one expects this stress to influence snails mostly during the November-to-April rainy and cold season. Species intolerant of prolonged summer drought and heat during the May-to-October period of aestivation should have slower growth and be more developmentally unstable on the ‘African’ slope, while those intolerant of shade and lower winter temperatures should have slower growth and be more developmentally unstable on the ‘European’ slope.

## Materials and Methods

### Site descriptions

‘Evolution Canyon I’ (EC I) (Figure 1) is located at Lower Nahal Oren (32°42′51.09″N; 34°58′26.81″E), a deeply incised valley running from Mount Carmel, Israel, westwards into the Mediterranean Sea. The opposite slopes share identical geological history (Plio-Pleistocene canyon, presumably 3–5 million years old [7]), geology, soils (terra rossa on Upper Cenomanian limestone), and regional climate, although they differ in topography (dip in opposite directions; the ‘African’ slope dips 35°; the ‘European’ slope dips 25°) and aspect. Interslope distance is 100 m at the valley bottom and 400 m at the top; ‘African’ and ‘European’ slopes are 120 m and 180 m long, respectively (Figure 1). Rainfall at ‘Evolution Canyon I’ is 600 mm per year. The percentage of plant cover varies from 35% on the ‘African’ slope to 150% on the ‘European’ slope [19]. Life-form analysis clearly illustrates the dramatic interslope differences between the hot, xeric, Mediterranean savannoid formation of *Ceratonia siliqua–Pistacia lentiscus* on the ‘African’ slope and the dense maquis of *Quercus calliprinos–Pistacia palaestina* on the ‘European’ slope [19].

‘Evolution Canyon II’ (EC II) is located 38 km northeast of ‘Evolution Canyon I’ at Lower Nahal Keziv, western Upper Galilee (33°02′34.86″N, 35°11′05.74″E). Like ‘Evolution Canyon I,’ ‘Evolution Canyon II’ has a south-facing ‘African’ slope and a north-facing ‘European’ slope that incline 20–40° and 30–40°, respectively. The canyon is narrower and steeper than that at ‘Evolution Canyon I’ (50 m at the bottom and 350 m at the top). It is also further inland from the Mediterranean Sea, and more sheltered, than ‘Evolution Canyon I.’ The underlying rocks are upper Cenomanian limestone, with colluvial and alluvial soils at the bottom and terra rossa on the slopes. Rainfall at ‘Evolution Canyon II’ is 700 mm per year, which is 17% greater than that at ‘Evolution Canyon I.’ The plant communities also vary between the slopes. The number of vascular plant species on the ‘African’ slope (205 species) is substantially greater than on the ‘European’ slope (54 species). The percentage of plant cover varies from 70% on the ‘African’ slope to 100% on the ‘European’ slope [20]. The ‘African’ slope changes from *Calicotome villosa* and *Salvia fruticosa* garrigue at the bottom to a dry, Mediterranean, savannoid, open Park Forest of *C. siliqua* – *P. lentiscus* association at the top. The ‘European’ slope is covered by a dense forest of *Acer obtusifolium* and *Laurus nobilis*, which is very different from the ‘European’ slope of ‘Evolution Canyon I,’ and represents a Mediterranean maquis forest.

### Sampling

We collected shells of six species of shelled gastropods (both juveniles and adults) from north- and south-facing slopes of ‘Evolution Canyon I’ and ‘Evolution Canyon II’ (Table 1). The collections were approved by the Israeli Nature and Park Authority [Permit 2010/38005 and 2010/38006 for Oren Canyon (‘Evolution Canyon I’) and Keziv Canyon (‘Evolution Canyon II’), respectively], so all necessary permits were obtained for the described field studies.

**Table 1:** Species of snails at ‘Evolution Canyons I and II’ used in this study. Diet and habitat descriptions are from Pavlíček *et al.* [13].

Family	Species	Diet	Habitat
Pomatiidae	*Pomatias olivieri* (de Charpentier, 1847)	Decaying plants	Shade
Enidae	*Buliminus labrosus labrosus* (Olivier, 1804)	Lichens, liverworts	Rocky outcrops
Oxychilidae	*Eopolita protensa jebusitica* (Roth, 1855)	Invertebrates, decaying plants	Under stones, wood, and leaves
Hygromiidae	*Monacha syriaca* (Ehrenberg, 1831)	Green plants	Garrigue, terraces
	*Xeropicta vestalis joppensis* (Schmidt, 1855)	Green plants	Garrigue, terraces
Helicidae	*Levantina spiriplana caesareana* (Mousson, 1854)	Lichens, liverworts	Rocky outcrops

We sampled four land-snail species from the opposing slopes of ‘Evolution Canyon I,’ the pulmonate snails *Buliminus labrosus labrosus*, *Monacha syriaca*, *Xeropicta vestalis joppensis*, and *Levantina spiriplana caesareana*. Two of these species, *L. s. caesareana* and *B. l. labrosus*, were also sampled at ‘Evolution Canyon II.’ In addition, we sampled *Pomatias olivieri*, a prosobranch snail, and *Eopolita p. jebusitica*, a pulmonate snail, only at ‘Evolution Canyon II.’ The taxonomy of *Buliminus*, *Monacha*, *Xeropicta*, *Pomatias*, and *Eopolita* follows Heller [21], while *Levantina* follows Pfeiffer [22] and Forcart (unpublished work).

### Measurements

We scanned each snail twice on a flatbed scanner, at a resolution of 600 dpi. To support a snail for the scan, we pressed it into a cubic block of clay so that the columella was either parallel or perpendicular to the scan surface, depending upon the species. For replicate scans, and to estimate the measurement error associated with each scan, we repositioned each snail in the clay, from scratch. We also made three replicate sets of measurements per scan, using SigmaScan Pro: Image Analysis Version 5.0.0. Consequently, there were six replicate measurements made on each snail. The main measurement was the radius from apex to curve (the suture) for every 180° of clockwise rotation. All scans were done by Shmuel Raz. All measurements on the images were made by a single observer (Nathan Schwartz).

Different species of snails required different approaches. Those having a relatively depressed, flat shell (*Monacha*, *Xeropicta*, *Eopolita*, and *Levantina*) could be scanned such that the apex and entire spiral suture were clearly visible (apical view, with columella perpendicular to the scan surface). Measuring the radius from apex to curve of the suture was straightforward. This could not be done with snails having an oblong or globose shell (*Pomatias* and *Buliminus*). These species were scanned from the side, in apertural view (columella parallel to the scan surface). We measured the distance from apex to the nearest suture on the left side, and then from that suture to the next one, and so on, repeating the process on the right side.

### Fluctuating helical asymmetry

**Figure 2:**
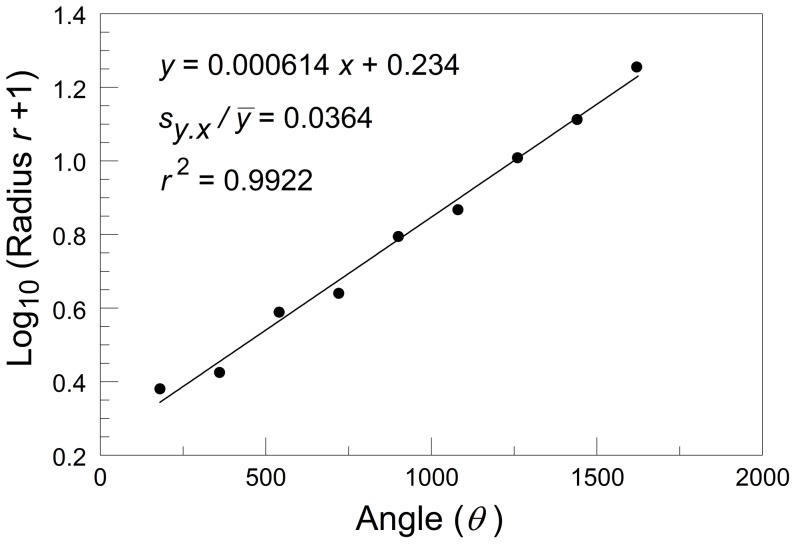
Linear regression of the log_e_ of the radius *r*+1 on the angle of rotation in degrees, for an individual *Buliminus l. labrosus* from the ‘African’ slope of ‘Evolution Canyon II.’

Helical symmetry involves rotation, along with translation along an axis of rotation. The spiral shell approximates an equiangular (logarithmic) spiral. The equation for an equiangular spiral is *r* = *ae^θ^* ^cot *Φ*^, where *r* is the radius from apex to curve, *a* is a constant, *e* is the base of natural logarithms, *θ* is the angle made with a reference line passing through the apex, and *Φ* is the constant angle at which the radius vector cuts the curve. Graham *et al.* [2] regressed log_e_ (*r*+1) on angle *θ* for each individual snail and used the standard error of the estimate, divided by the mean of the dependent variable (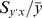), as an estimate of individual asymmetry (Figure 2).

Measurement error (*s* ^2^_me_) inflates estimates of fluctuating asymmetry. It also creates problems when the researcher later corrects for size scaling [1], [23], [24]. A preliminary study of *Cepaea nemoralis* (previously collected in the Ukraine) suggested that the variation among photos within snails within sites accounted for 22.5% of the variation, while variation among replicate measurements accounted for 3.5%. The remaining variation (74.0%) was among individual snails within a site.

Trait-size variation is often a problem in studies of fluctuating asymmetry. Positive size-scaling of asymmetry, for example, is largely due to multiplicative error associated with the active-tissue model of growth [1], [24]. We found no evidence for positive (or negative) size scaling after averaging all of the replicate measurements and log_e_ transforming *r*. The averaging of replicates removes most of the additive measurement error and the logarithmic transform eliminates the multiplicative error associated with growth.

### Size and growth

As a measure of body size, we measured the height of the shell from the apex to the closest part of the aperture and quantified the rate of expansion of the body whorl as the slope of the regression of log_e_ (*r*+1) on angle *θ*. The number of complete rotations of the suture around the apex is also an indicator of size. This is not equivalent to the number of whorls; the number of suture rotations always exceeds the number of whorls.

### Statistical analysis

We used SPSS's GLM Varcomp procedure to estimate the variance components associated with sites, snails within sites, scans of snails within sites, and replicate measures of scans within snails within sites.

We used one-way ANOVA to compare fluctuating asymmetry, shell height, regression coefficient, and number of rotations of the suture between ‘African’ and ‘European’ slopes. Slope is a fixed effect and snail within slope (the average of six replicate measurements) is a random effect. For *Levantina* and *Buliminus*, which were sampled at both ‘Evolution Canyons I and II,’ we included canyon as a fixed effect.

## Results

### Fluctuating helical asymmetry

Variance components associated with slope, individuals, scans, and replications were estimated for *B. l. labrosus* and *L. s. caesareana* (Table 2). The among-individual variation represents both genotypic and microenvironmental variation. Measurement error includes variation among scans and among replicate measurements. For *Levantina*, which we scanned in apical view, most of the variation was due to measurement error (49–57% of the total variation was among scans and 17–30% was among replicate measurements). For *Buliminus*, which we scanned in apertural view, measurement error was much smaller (4% of the total variation was among scans and 1% was among replicate measurements).

**Table 2:** Variance components for shell radii: *s* ^2^_slope_ is the between slope variation, *s* ^2^_ind_ is the among-individual variation, *s* ^2^_scan_ is the among scans variation, *s* ^2^_ repl_ is the variance component associated with replication, and *s* ^2^_me_ is the sum of *s* ^2^_scan_ and *s* ^2^_ repl_.

Variance component	*Levantina* (EC I)	*Levantina* (EC II)	*Buliminus* (EC I)
*s* ^2^_slope_	0.00009670	0.00015303	0.00003503
*s* ^2^_ind_	0.00063522	0.00008164	0.00304639
*s* ^2^_scan_	0.00103574	0.00100532	0.00013466
*s* ^2^_ repl_	0.00035143	0.00052515	0.00003247
*s* ^2^_me_	0.001387	0.001530	0.000167

**Figure 3:**
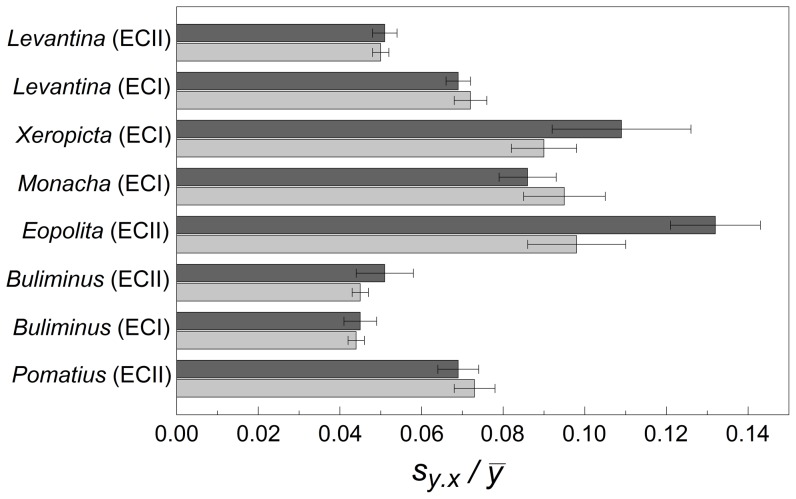
Mean fluctuating helical asymmetry (± standard error) of snails on ‘African’ and ‘European’ slopes. Light gray indicates the ‘African’ slope; dark gray indicates the ‘European’ slope.

*Eopolita p. jebusitica* at ‘Evolution Canyon II’ showed marginally significant differences in fluctuating asymmetry between the ‘African’ and ‘European’ slopes (*F*_1,__18_ = 4.146, *P* = 0.057, Figure 3). Shells were more asymmetric on the ‘European’ slope. None of the other species showed significant differences in fluctuating asymmetry between the slopes (*F*_1,__22–93_≤1.19, *P*≥0.215).

*Levantina s. caesareana* had greater fluctuating asymmetry at ‘Evolution Canyon I’ than at ‘Evolution Canyon II’ (*F*_1,__145_ = 36.978, *P*<0.001). Neither the differences between slopes (*F*_1,__145_ = 0.031, *P*>0.850), nor the interaction of slope and canyon (*F*_1,__145_ = 0.279, *P*>0.550) were significant. *Buliminus l. labrosus*, the only other species collected at both canyons showed no differences in fluctuating asymmetry between them (*F*_1,__158_ = 0.813, *P*>0.350).

### Shell height

**Figure 4:**
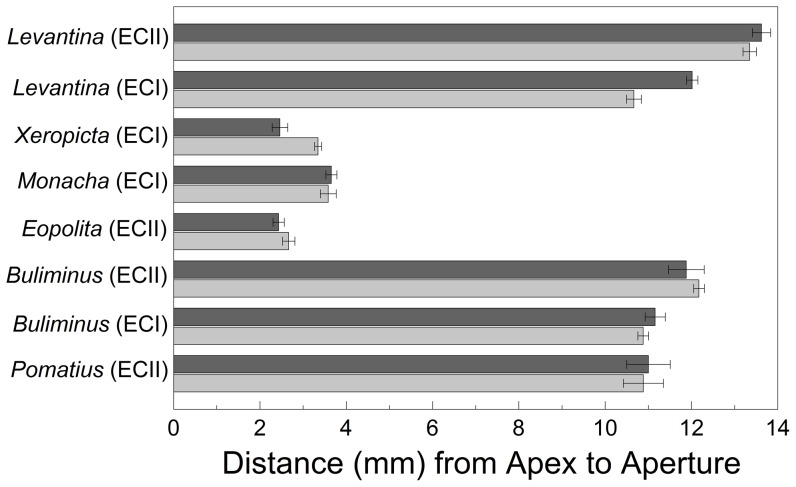
Mean shell height of snails (± standard error), from apex to aperture, on ‘African’ and ‘European’ slopes. Light gray indicates the ‘African’ slope; dark gray indicates the ‘European’ slope.

Mean shell height is indicative of overall size (Figure 4). There were significant differences in shell height between *L. s. caesareana* from the two canyon sites (*F*_1, 145_ = 150.414, *P*<0.001) and from ‘African’ and ‘European’ slopes (*F*_1, 145_ = 21.117, *P*<0.001). There was also a significant interaction between canyon site and slope (*F*_1, 145_ = 9.338, *P*<0.005). Shell heights were greater on the ‘European’ slope, though the differences were less extreme at ‘Evolution Canyon II,’ and shell heights were greater at ‘Evolution Canyon II’ than at ‘Evolution Canyon I.’ *Xeropicta v. joppensis*, in contrast, had greater shell height on the ‘African’ slope (*F*_1, 38_ = 19.811, *P*<0.001).

There were significant differences in shell height between *B. l. labrosus* from the two canyon sites (*F*_1, 158_ = 15.916, *P*<0.001), but not between ‘African’ and ‘European’ slopes (*F*_1, 158_ = 0.001, *P*>0.950). The interaction between canyon site and slope was also insignificant (*F*_1, 158_ = 1.280, *P*>0.250). Shell heights were greater at ‘Evolution Canyon II’ than at ‘Evolution Canyon I.’

The shell heights of *M. syriaca*, *P. olivieri*, and *E. p. jebusitica* did not differ between the two slopes (*F*_1, 18–32_≤1.409, *P*≥0.251).

### Expansion of the body whorl

**Figure 5:**
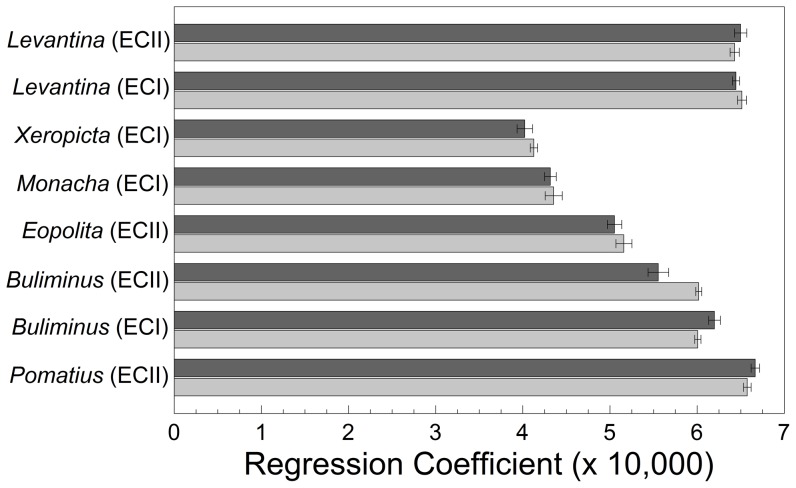
Mean regression coefficient (×10,000) (± standard error) on ‘African’ and ‘European’ slopes. Light gray indicates the ‘African’ slope; dark gray indicates the ‘European’ slope.

The rate of expansion of the body whorl (i.e., the slope of the regression of log_e_ *r*+1 on angle *θ* for each individual) reflects the rate at which the spiral opens up (Figure 5). Only *B. l. labrosus* showed significant differences between the canyon sites (*F*_1, 158_ = 19.560, *P*<0.001) and marginally significant differences between the two slopes (*F*_1, 158_ = 3.682, *P* = 0.057). The interaction between site and slope was also significant (*F*_1, 158_ = 20.870, *P*<0.001). The body whorl expanded more rapidly on the ‘European’ slope at ‘Evolution Canyon I,’ but the reverse was true at ‘Evolution Canyon II.’ None of the other species displayed differences in the body whorl between the ‘African’ and ‘European’ slopes (*F*_1, 18–93_ = 0.765–2.050, *P*≥0.166).

### Number of rotations of the body suture

**Figure 6:**
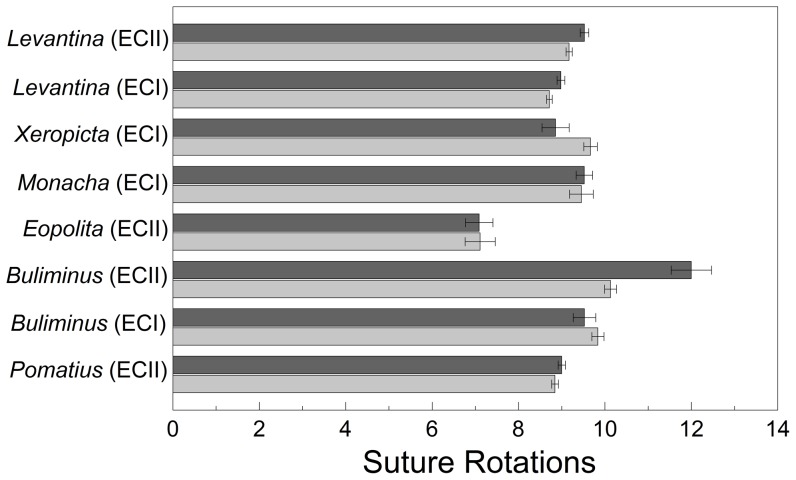
Mean number of rotations of the suture (± standard error) on ‘African’ and ‘European’ slopes. Light gray indicates the ‘African’ slope; dark gray indicates the ‘European’ slope.

The mean number of rotations of the body suture (Figure 6) is indicative of age and size. Populations of *B. l. labrosus* at ‘Evolution Canyon II’ had more suture rotations on the ‘European’ slope than on the ‘African’ slope (*F*_1, 73_ = 12.374, *P*<0.001). Populations of *L. s. caesareana* had significant differences in the number of suture rotations between the two canyon sites (*F*_1, 145_ = 48.126, *P*<0.001) and between ‘African’ and ‘European’ slopes (*F*_1, 145_ = 18.731, *P*<0.001). The interaction between canyon site and slope was insignificant (*F*_1, 145_ = 0.355, *P*>0.550). The number of rotations of the body suture was greater on the ‘European’ slope.

There were significant differences in the numbers of suture rotations between *Buliminus* from the two canyon sites (*F*_1, 158_ = 26.637, *P*<0.001), and from ‘African’ and ‘European’ slopes (*F*_1, 158_ = 7.497, *P*<0.010). The interaction between canyon site and slope was also significant (*F*_1, 158_ = 14.704, *P*<0.001). The number of suture rotations was greater at ‘Evolution Canyon II’ than at ‘Evolution Canyon I,’ but differences between ‘African’ and ‘European’ slopes were only evident at ‘Evolution Canyon II,’ where snails on the ‘European’ slope had more suture rotations.

In contrast to *Buliminus* and *Levantina*, *X. v. joppensis* had more suture rotations on the ‘African’ slope (*F*_1, 38_ = 5.492, *P*<0.025). None of the other species and populations had different numbers of suture rotations on the two slopes (*F*_1, 18–85_ = 0.002–1.833, *P*≥0.189).

## Discussion

Gastropods are distributed from the arctic to the tropics and can be terrestrial (one-third of species) or aquatic (two-thirds of species) [25]. They are adapted to most of the habitats on Earth and in terrestrial habitats they are subjected to daily and seasonal variation in temperature and water availability. Their success in colonizing different habitats is due to physiological, behavioral, and morphological adaptations to water availability [26], [27], [28], as well as ionic and thermal balance [29]. The shell of a snail is constructed of calcium carbonate, but even in acidic soils one can find various species of shell-less slugs. Interestingly, land-snails also live in deserts, where they must contend with heat and aridity [30].

### Species richness and abundance

Adaptation of land-snails to different regimes of heat and aridity may influence species richness and abundance of snails on the opposing slopes of ‘Evolution Canyon’ [13], [17], [27], [31], [32]. For example, species richness is greater on the ‘European’ slope of ‘Evolution Canyon I,’ but overall abundance is greater on the ‘African’ slope [13]. The greater species richness on the ‘European’ slope represents the addition of European species of snails and slugs at the southern limits of their adaptive range.

The greater overall abundance of snails on the ‘African’ slopes may reflect a better nutritional environment, less predation from small mammals [33], abiotic stress or all of these together. Early successional plants on the ‘African’ slope may be more palatable [34], and plant secondary compounds influence choice of food [35]. If this is true, then the snails that feed on live plants and lichens should show the greatest differences in abundance. The four species that feed on living plants or lichens (*L. s. caesareana*, *B. l. labrosus*, *M. syriaca*, *X. v. joppensis*) were rare (*M. syriaca*) or considerably more abundant on the ‘African’ slope, whereas the species that feed on decaying plants (*P. olivieri*, *E. p. jebusitica*) or other invertebrates (*E. p. jebusitica*) were slightly more abundant on the ‘European’ slope.

According to Pavlíček *et al.* [13], six of seven species more abundant on the south-facing slope fed on live plants, whereas only one of eleven species that fed exclusively on decaying plants was more abundant on the south-facing slope. Finally, the main predators, such as shrews (*Crocidura* spp. and *Suncus etruscus*), may be unwilling to venture out onto the more open south-facing slopes, which lack natural shelters [36].

### Fluctuating helical asymmetry

To the best of our knowledge, the only work on fluctuating helical asymmetry of land-snails was done by Graham *et al.* [2], who studied deviations from a perfect equiangular spiral in three populations of the terrestrial land-snail *Cepaea nemoralis* in the Ukraine. The highest level of asymmetry was found in populations exposed to ammonia emissions and pesticides. Individuals in the population having the greatest helical asymmetry also showed erosion of their periostracum, which was not evident in the other two populations.

Overcrowding and nutritional deprivation can influence shell microstructure, increasing fluctuating helical asymmetry. Chunhabundit *et al.* [3], for example, raised maculated top shells, *Trochus maculatus*, a marine gastropod, under high density and inadequate nutrition. The periostracum was reduced and shell structure was dissolved in the vicinity of the shell apex. The suture lines were less smooth.

As with twelve species of vascular plants at ‘Evolution Canyon I,’ we cannot reject the null hypothesis of no differences in deviations from perfect symmetry between the land-snails from the opposing slopes. In the case of the vascular plants, the leaves were bilaterally symmetrical. For the snails, the shells are helically symmetrical. We suggest that land-snails are better adapted to the ‘African’ slope than we had anticipated. They can hide themselves in different locations, such as under stones and in cracks. Such behavior reduces their temperature and water loss, though *Xeropicta* aestivates high up on shrubs. In addition, the shells may serve as a CaCO_3_ ‘door,’ separating land-snails from environmental stress.

### Thermoregulation, drought resistance, and body size

There is a trade-off between drought resistance and thermoregulation in snails [32]. Thermoregulation requires water for evaporative cooling. The smaller the snail, the more effective the cooling, but this system nevertheless requires more water. Evaporative cooling is more effective for smaller snails because a smaller body size increases the surface area to volume ratio, which in turn increases both absorption and radiation of heat [32], [37]. The ecological rule associated with this phenomenon is Bergmann's rule [38], which predicts larger body size of warm-blooded vertebrates in colder areas. According to Mayr [39], this is an adaptive response to environmental temperatures. Although Bergmann's rule was intended to describe body-size variation among species, it has been extended to intraspecific variation in body size [39], [40] as well as to ectotherms [41], but with variable success [42], [43]. This rule is exemplified by the spiny mouse, *Acomys cahirinus*, and the broad-toothed field mouse, *Apodemus mystacinus*, from the opposing slopes of ‘Evolution Canyon.’ Individuals from the ‘African’ slope are smaller [44] than those from the ‘European’ slope.

Is it possible that Bergmann's rule, which was meant for endothermic species, also holds for land-snails? Are the size differences of the snails between the canyons and the opposing slopes a result of microclimatic differences? We believe that, yes, the larger shells of *Levantina s. caesareana* at ‘Evolution Canyon II’ (i.e., Keziv Canyon) and on the ‘European’ slopes of both canyons do indeed represent Bergmann's rule on a microscale. Nevertheless, we collected both adults and juveniles, hence size differences may also be due to differences in age structure.

These results are supported by evidence for a correlation between habitat and body size in invertebrates. The body size of insects and spiders, for example, is smaller when the humidity is lower [45], [46]. Moreover, a correlation was found between shell diameter and climate in the snail *Xerocrassa seetzenii*, from Israel [47]. The latitudinal gradient of decreasing body size, from north to south, in Israel, accompanied by decreasing rainfall, also occurs in *Levantina s. caesareana* [48].

The smaller the individual, however, the more water it loses for cooling. Hence, there is a lower-size threshold for a given individual and environment [49]. Very small animals lose almost 100% of their body mass for cooling [50]. Accordingly, the amount of water in the tissues of *X. v. joppensis* may be so small that they are selected for larger body size on the ‘African’ slope than on the ‘European’ slope of ‘Evolution Canyon.’

*Xeropicta* lives for only one year, while *Levantina* and *Buliminus* live for several years. During years with reduced rainfall, adult *Xeropicta* and *Monacha* may be extremely small, while in a year with extremely high rainfall and numerous nights with heavy dew, they may reach very large size (Mienis, personal observations).

Morphological differences in body size between relatively large snails (*Buliminus* and *Levantina*) and somewhat smaller snails (*Monacha*) from the opposing slopes of ‘Evolution Canyon’ were found in previous studies [15]. Accordingly, five-out-of-seven species were larger on the ‘European’ slope.

## Conclusions

The differences in fluctuating helical asymmetry between ‘African’ and ‘European’ slopes were either non-existent or only marginally significant (shell asymmetry of *E. p. jebusitica* was marginally greater on the north-facing slope), hence we cannot reject the null hypothesis for no differences between the slopes. There were, however, differences between shell asymmetry of *L. s. caesareana* from the two canyons; asymmetry was greater at the more arid Nahal Oren, ‘Evolution Canyon I.’
